# Title Expression of FOXO3 and MAPK1 Genes in Patients with Benign Salivary Gland Tumors

**DOI:** 10.3390/jcm13010215

**Published:** 2023-12-29

**Authors:** Katarzyna Kolary-Siekierska, Piotr Niewiadomski, Wojciech Namieciński, Jarosław Miłoński

**Affiliations:** Department of Otolaryngology and Laryngological Oncology, Audiology and Phoniatrics, Medical University of Lodz, 90-549 Lodz, Poland

**Keywords:** pleomorphic adenoma, Warthin tumor, MAPK1, FOXO3, gene expression

## Abstract

Pleomorphic adenomas (PAs) and Warthin tumors (WTs) are the most common benign tumors that occur in the salivary gland. PA has a tendency towards malignant transformation. Thus, searching for new methods to diagnose salivary gland tumors and treatment is important. The members of the class O forehead box transcription factor (FOXO3) and mitogen-activated protein kinase 1 (MAPK1) genes participate in the cellular processes, including in cell proliferation. The aim of this study was to analyze these genes’ expression in the salivary gland tissues and in salivary gland tumors. The study group consisted of 50 patients treated for salivary gland tumors. For genetic tests, fresh samples of tissue collected during the surgery were used. The expression levels of the FOXO3 and MAPK1 genes were statistically significantly lower in PA tissue than in normal salivary gland tissue and WT tissue. This research revealed that the FOXO3 and MAPK1 genes are present in benign salivary gland tumors and also indicated a role of these genes in the development of benign salivary gland tumors. The cause of the development of pleomorphic adenomas may be apoptotic disorder and the activation of the inflammatory process. The examined genes may have potential to be new therapeutic targets for the treatment of pleomorphic adenomas.

## 1. Introduction

Pleomorphic adenomas (PAs) and Warthin tumors (WTs) are the most common benign tumors that occur in the parotid gland. PAs accounts for 60% to 70% of cases, the second most common type of tumor is WTs. WTs mostly develop in patients between 50 and 70 years of age (average age of 60 years) [1] and have been strongly connected with smoking. The etiology of PAs is still unknown. A few of the causes may be exposure to radiation, chemicals, simian virus 40 (SV 40), smoking, or a genetical predisposition (the chromosomal aberration of 8q12 and 12q15) [2]. PAs develop most often in patients between 30 and 60 years of age (average age of 47 years). The incidence of neoplasm in a women: men ratio is 1:1.4 to 1:1.7 [3]. The characteristic features of PAs are pseudopodia and satellite nodules, which may infiltrate the salivary gland tissues and provoke neoplasm recurrence [4,5]. The risk of transformation of PAs into carcinoma ex pleomorphic adenomas (CXPA) rises to 9.5% after 15 years. CXPA accounts for about 3 to 5.7% of all salivary gland tumors [6].

The members of class O forkhead box transcription factor 3 (FOXO3) are also known as FOXO2, AF6q2, FKHRL1, FOXO3A, and FKHRL1P2. FOXOs are placed in the nucleus, where they function as transcription factors. They affect the expression of the genes that are necessary to induce the apoptosis process, DNA damage repair, differentiation, and cell-cycle arrest. They may also influence the regulation of glucose metabolism in the organs and resistance to oxidative stress. The transcriptional activity is regulated via acetylation, deacetylation, ubiquitination, and arginine or lysine methylation. They may inhibit the cell cycle via p27 KIP1 and p21 WAF1 gene expression and promote apoptosis via Fas ligand, Bim, and TRAIL gene expression. They may decrease oxidative stress via superoxide dismutase and catalase gene expression. FOXO3 genes act also as tumor suppressors [7,8]. In response to stress, FOXO3 has a role in proteostasis, autophagy, mitophagy, lipophagy, gluconeogenesis, immune system activity, the regulation of cell death, and the redox balance process. These cellular processes protect against diabetes type 2, cardiovascular disease, cancer, and neurological disorders and have an influence on longevity [9].

Mitogen-activated protein kinases (MAPKs) are also known as extracellular-signal-regulated kinases (ERKs). The MAPK 1 gene, known as ERK, p38, p40, p41, ERK2, ERT1, ERK-2, MAPK2, PRKM, PRKM2, P42MAPK, p41mapk, and p42-MAPK, is located on chromosome 22q11.22. Mitogen-activated protein kinases perform many functions in the cell, including differentiation, proliferation, apoptosis, and the inflammatory response. The ERK1 and ERK2 kinases may influence signal transmission from the cell surface into the cell and are involved in many cell processes, including cell adhesion, migration, proliferation, differentiation, and the maintenance of the cell cycle. These kinases may play an important role in the development of the following human cancers: breast cancer, hepatocellular cancer, lung cancer, colorectal cancer, and pancreatic cancer [10]. The MAPK/ERK1 kinases (encoded by MAPK1) can phosphorylate the FOXO3a molecule (encoded by the FOXO3 gene), and as a result of this, FOXO3a moves to the cytoplasm of the cell and, after ubiquitination with the participation of MDM2, is proteosomally degraded [11,12]. FOXO3 degradation affects cell survival by inhibiting apoptosis [11,13].

Searching for new methods to diagnose and treat salivary gland tumors is of vital importance. Gene expression may prove to be useful. So far, the studied genes in pleomorphic adenomas and Warthin tumors have not been described. The aim of this study was to analyze FOXO3 and MAPK1 gene expression in the salivary gland tissues and salivary gland tumors.

## 2. Materials and Methods

### 2.1. Study Participants

The patients were from the Department of Otolaryngology, Laryngological Oncology, Audiology and Phoniatrics at the Medical University Teaching Hospital No. 2 in Lodz. The patients gave informed and voluntary consent to the procedure and participation in the study. This study was conducted in accordance with the Declaration of Helsinki and was approved by the Institutional Review Board (or Ethics Committee) of the Medical University of Lodz (protocol code RNN/222/17/KE + KE/408/20).

The study group consisted of 50 patients who were treated for salivary gland tumors (25 Warthin tumors, 25 pleomorphic adenomas) and were aged 23 to 80 years (mean age of 58.45 years) from whom a fragment of tumor was collected. WHO 2022 classification to assess tumors was used. As part of preoperative diagnosis, a history and physical examination, laboratory tests (morphology, creatinine, and CRP), and additional tests were performed: ultrasound-guided FNAB examination, CT, or MRI with contrast. In all cases, the following surgical treatment was performed: partial parotidectomy with preservation of the facial nerve.

Survey data, gender, age, weight, height, chronic diseases (diabetes, hypertension, hypercholesterolemia), and information regarding smoking and alcohol use, were obtained from all study participants. Patients answered questions about smoking cigarettes. If they smoked, they also answered questions about period of smoking and amount of smoked cigarettes per day. Their body mass index (BMI) was calculated according to the following formula: weight [kg]/height [cm]^2^. Based on the BMI criteria according to the World Health Organization (WHO), which indicates that the normal body weight is a BMI in the range of 18.5–24.99, body mass index > 25 indicates overweight, and >30 indicates obesity. Characteristic features of the investigated group are described in Table 1.

The control group included the same patients from whom a fragment of normal salivary gland tissue was collected. Samples of tissue were collected during partial parotidectomy. A piece of normal salivary gland tissue was taken from the further part of the tumor and a macroscopically unchanged salivary gland. Histopathological examination confirmed normal salivary gland tissue at the site of material collection.

### 2.2. Collection of Samples

The removed tumors were subjected to histopathological examination and were assessed according to the WHO 2022 classification of head and neck cancers.

For genetic tests, a piece of tissue (tumor and normal salivary gland tissues) collected during surgery and fixed in RNAlater Solution (Qiagen, Hilden, Germany) was used. RNAlater Solution prevents RNA degradation. Each sample was evaluated by the pathologist. The tumor fragment was placed in Eppendorf tubes with 1 mL of RNAlater fluid (Qiagen, Hilden, Germany) and was frozen at −20 °C. Then, it was sent to the Central Laboratory of the Medical University of Lodz to evaluate the expression of FOXO3 and MAPK1 genes. Then, expression of the chosen genes was estimated via appropriate molecular techniques as described below.

### 2.3. Total RNA Extraction

Total RNA from fresh tissues stabilized with RNAlater Solution (Qiagen, Hilden, Germany) was isolated using the RNeasy Mini Kit (Qiagen, Hilden, Germany) according to the manufacturer’s instructions. Tissues were homogenized in 350 µL of QIAzol Lysis Reagent using a TissueRuptor homogenizer (Qiagen, Hilden, Germany) and were then centrifuged for 3 min. at 16,000× *g*. An equal volume of 70% ethanol was added to the supernatant, and this mixture was applied to columns for purification. The columns were washed with 700 µL of RW1 buffer and twice with 500 µL of RPE buffer. The purified RNA was eluted with 30 µL of RNase-free water.

The yield and quality of the purified RNA was determined using a PicoDrop spectrophotometer (Picodrop Limited, Radnor, PA, USA), determining an absorbance ratio of 260 nm/280 nm. For further tests, samples with absorption coefficient A260/280 > 1.8 were used. Purified total RNA was either used immediately for cDNA synthesis or stored at −80 °C until use.

### 2.4. Reverse Transcription

A reverse transcription reaction was performed using Maxima First Strand cDNA Synthesis kit (Thermo Fisher Scientific Inc., Foster City, CA, USA) according to the manufacturer’s protocol. In total, 1 µg of total RNA was used for each reaction, and reverse transcription was performed under the following conditions: 25 °C for 10 min, 50 °C for 30 min, and 85 °C for 5 min. The cDNA samples were used for qPCR reactions or were stored frozen at −20 °C.

### 2.5. Real-Time PCR

Real-time polymerase chain reaction (qPCR) was performed with commercial TaqMan^®^ Gene Expression Assay kits. In the qPCR reaction, commercial TaqMan^®^ Gene Expression Assay kits (Thermo Fisher Scientific Inc.) were used for the following genes: the members of the class O forehead box transcription factors (FOXO3s, Hs00818121_m1), mitogen-activated protein kinase 1 (MAPK1, Hs01046830_m1), and glucuronidase beta (GUSB, Hs00939627_m1) as potential endogenous controls. Assays were performed in 96-well optical plates using a 7900HT Fast Real-Time PCR System (Thermo Fisher Scientific Inc.). For each qPCR reaction, 50 ng of cDNA, 0.5 µL of the appropriate TaqMan Gene Expression Assay, and 5 µL of TaqMan Universal Master Mix (Thermo Fisher Scientific Inc.) were used. The reaction was carried out in the volume of 10 μL under the following conditions: initial denaturation for 20 s at 95 °C and 40 cycles for 3 s at 95 °C and 30 s at 60 °C. The results were analyzed using the Sequence Detection System 2.4 software and Data Assist (Thermo Fisher Scientific Inc.) and presented as Cq or were calculated via ΔΔCt method values of the relative quantification of the expression (RQ).

### 2.6. Statistical Analysis

Statistica (version 8.1) was used for all statistical procedures. The normality of distribution was appraised by using Shapiro–Wilk test. The homogeneity of variances was checked by using Levene’s test. *p*-values < 0.05 were statistically significant.

**Figure 1:**
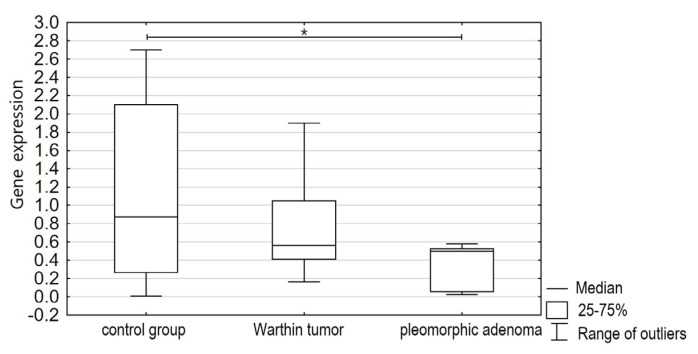
Comparison of FOXO3 gene expression in salivary gland tissue (control group) and salivary gland tumors—Warthin tumors and pleomorphic adenomas (study group), *p* < 0.05 (*).

**Figure 2:**
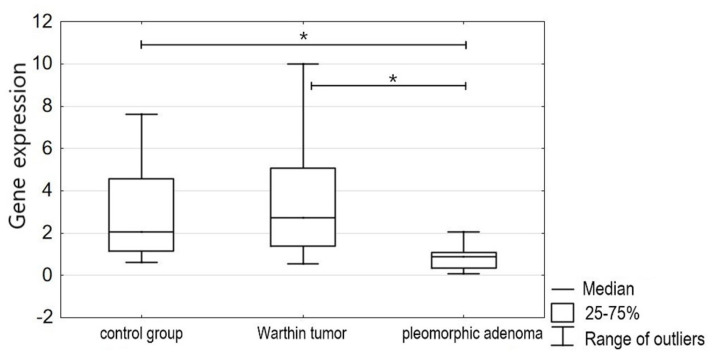
Comparison of MAPK1 gene expression in salivary gland tissue (control group) and salivary gland tumors—Warthin tumors and pleomorphic adenomas (study group), *p* < 0.05 (*).

Grubbs’ test, Levene’s test, Shapiro–Wilk test, and Wilcoxon’s test were used to assess genes expressions (Figure 1 and Figure 2) in Table 1 used Shapiro–Wilk test, Levene’s test, chi^2^ independent test, Student’s t-test, or U Manna–Whitney test. In Table 2, chi^2^ independent test was used. Eta squared test correlation coefficient was used to check correlation between gene expression and smoking cigarettes. Correlation between BMI and age of patients with gene expression was tested by using Spearman’s rank correlation coefficient.

## 3. Results

The members of the class O forehead box transcription factors’ (FOXO3s) gene expression was statistically significantly lower (*p* < 0.05) in pleomorphic adenoma tissues than in normal salivary gland tissues or in Warthin tumor tissues. The mean FOXO3 mRNA expression in the pleomorphic adenomas was 0.6496 (SD 0.72); that in the normal salivary gland tissues was 1.7234 (SD 2.8613); and that in the Warthin tumors was 2.0633 (SD 3.9353) (Figure 1).

The mitogen-activated protein kinase 1 (MAPK1) gene expression was statistically significantly lower (*p* < 0.05) in the pleomorphic adenoma tissues than in the normal salivary gland tissues or in the Warthin tumor tissues. The mean MAPK1 mRNA expression in the pleomorphic adenomas was 1.2671 (SD 1.1469); that in the normal salivary gland tissues was 3.5357 (SD 3.4062); and that in the Warthin tumors was 3.16 (SD 2.5721). The difference in the expression of the MAPK1 gene between the pleomorphic adenomas and Warthin tumors was also statistically significant (*p* < 0.05) (Figure 2).

The patients with PAs were younger than the patients with WTs (mean age of 54.2 vs. 67.7 years). The patients with WT often smoked cigarettes. They also smoked more cigarettes per day for a longer time than the patients with PAs. The characteristic features of the investigated group are shown in Table 1.

Most of the tumors were located in the parotid gland. They were soft in consistency and movable in relation to the ground. The first-choice treatment was a partial parotidectomy. The characteristic features of the tumors are shown in Table 2.

Our study showed that smoking cigarettes, the number of cigarettes per day, the years of smoking, BMI, and the age of patients were not statistically significant predictors of MAPK1 and FOXO3 expression in the study groups. The eta squared correlation coefficient for FOXO3 expression and smoking in the Warthin tumors was 0.0445, and that for the pleomorphic adenomas was 0.2396. The eta squared correlation coefficient for MAPK1 expression and smoking in the Warthin tumors was 0.1839, and that for the pleomorphic adenomas was 0.1352. Spearman’s rank correlation coefficient for FOXO3 expression and the age of the patients in the Warthin tumors was −0.0745, and that for the pleomorphic adenomas, respectively, was 0.2396. Spearman’s rank correlation coefficient for MAPK1 expression and the age of the patients in the Warthin tumors was 0.0578, and that for the pleomorphic adenomas, respectively, was 0.0278.

Spearman’s rank correlation coefficient for FOXO3 expression and BMI in the Warthin tumors was 0.019, and that for the pleomorphic adenomas, respectively, was −0.3159. Spearman’s rank correlation coefficient for MAPK1 expression and BMI in the Warthin tumors was 0.0883, and that for the pleomorphic adenomas, respectively, was −0.306.

## 4. Discussion

In malignant tumors, e.g., breast, intestinal, ovarian, prostate, liver, and nasopharyngeal cancers, MAPK/ERK pathway inhibitors are used to slow down disease progression [14]. There are many negative feedback loops in the MAPK/ERK pathway in cells; it turns out it is primarily the balance between the activating factors and inhibitors of this pathway that are important, not only the absolute level of MAPK/ERK activation. Moreover, some inhibitors of the pathway may contribute to development of neoplastic disease [15]. The deregulation of the ERK pathway is often connected with cancer development. Phosphorylated ERK-1/ERK-2 activation is associated with a more advanced stage of oligodendroglioma and the development of melanomas. They may also participate in metastasis in head and neck squamous cell carcinoma and in breast cancer. The ERK-1/ERK-2 pathway may contribute to tumorigenesis and the metastasis of salivary gland mucoepidermoid carcinoma. These pathways are an important target for new anticancer drugs [16]. Therapies with ERK inhibitors increase autophagy and the influence on the number, volume, and weight of liver metastases in pancreatic cancer [10]. ERK2 kinase (encoded by MAPK1) can phosphorylate the FOXO3a molecule (encoded by the FOXO3 gene), and as a result of this, FOXO3a moves to the cytoplasm of the cell and, after ubiquitination with the participation of MDM2, is proteosomally degraded [11,12]. FOXO3 degradation affects cell survival by inhibiting apoptosis [11,13]. In pancreatic cancer, MAPK/ERK kinase inhibitors are used to increase the activity of the FOXO protein in order to inhibit tumor progression [17]. The importance of FOXO3 in oral squamous cell carcinoma [18] as well as in liver cancer [19] was noted. In clear cell renal cell carcinoma, low levels of FOXO3 mRNA were observed to be an independent prognostic factor for metastases based on a multivariate Cox regression analysis. This finding suggests the possibility of using FOXO as a potential marker to select patients at high risk of metastases [20]. In breast cancer, FOXO3 overexpression was shown to be associated with tumor suppression [13,21,22]. FOXO proteins are transcription factors used to regulate cellular processes in response to changes in the external and internal environment. The downregulation of FOXO3a in mice affected fertility and the presence of ovarian follicle abnormalities [23]. Decreased FOXO3a activity is observed in urothelial carcinoma [24]. In colon cancer, it has been proven that FOXO3a and p38 are markers of response to cetuximab treatment [25]. FOXO3 also takes part into the induction and regulation of autophagy. Autophagy is a crucial process that is essential for cell survival in eukaryotic organisms. FOXO3 is responsible for two main processes: the ubiquitin proteasomal pathway and the autophagic lysosomal pathway [26]. FOXO3 has a fundamental role in the regulatory pathway of autophagy in the skeletal muscle in vivo. Mammucari et al. observed the overexpression of FOXO3 in skeletal muscle cells and in atrophic muscle fibers. The autophagy process is also important for the treatment of other diseases, including cancer [27]. FOXO influences cell-cycle inhibition and causes an increase in oxidative stress resistance. This protein shifts anabolic processes towards catabolic processes in cellular metabolism. Eijkelenboom et al. noticed that a relatively high FOXO level occurs in cells under normal conditions. FOXOs are involved in the maintenance homeostatic regulators in response to environmental changes, e.g., removing damaged cells because damaged cells may become tumor cells; thus, one of FOXO’s functions is tumor suppression. Moreover, FOXO may also take part in the homeostatic regulators in tumor cells and influence the progression of cancer [28]. A lower expression of FOXO3a was also associated with a reduction in the neural stem-cell pool [29] and the development of extensive inflammation. A decrease in FOXO3a expression resulted in the activation of the Th1 and Th2 cell responses. Mild inflammation was observed in mice, mainly in the salivary glands [30]. A FOXO gene deletion, e.g., the FOXO3a gene, may also affect the development of thymic lymphomas and angiomas, e.g., the liver (due to the inhibition of endothelial cell activity). In the thymocytes in which a complete loss of the FOXO gene’s function was observed, cell proliferation and survival increased, resulting in the development of lymphomas [31].

So far, the studied genes in benign salivary gland tumors have not been described. The expression of the FOXO and MAPK1 genes in pleomorphic adenomas and Warthin tumors was identified for the first time.

To conclude, the FOXO3 and MAPK1 genes are significant for maintenance homeostasis in normal cells. Generally, the overexpression of these genes causes a better outcome in the case of carcinomas. A decrease in the FOXO3 and MAPK1 genes’ expression inhibits the apoptosis and autophagy processes and shows an impact on inflammation development. Our study found a drop in gene expression in pleomorphic adenomas, which may suggest that these processes may be involved in this type of tumor development. The FOXO3 and MAPK1 genes may have potential as new markers and therapeutic targets in the treatment of pleomorphic adenomas. Nowadays, there is a trend to use (if possible) noninvasive operative techniques or to replace them with pharmacological therapy (e.g., a biological treatment).

Traditional anticancer drugs, like paclitaxel, cisplatin, imatinib, and doxorubicin, have an influence on FOXO3a activation [18]. This may lead, in the future, to the use of drugs acting on the described pathway to eradicate the tumors.

However, further studies of the molecular pathway involving a larger number of patients are necessary to be sure whether the studied genes have an impact on the development of pleomorphic adenomas.

## 5. Conclusions

This research revealed that FOXO3 and MAPK1 are presence in the salivary gland tissue and in benign salivary gland tumors and also indicated a role of these genes in the development of pleomorphic adenomas. The cause of the development of pleomorphic adenomas may be apoptotic disorder and the activation of the inflammatory process.

## Figures and Tables

**Table 1:** Characteristic features of the investigated group.

Analyzed Trait	Warthin Tumor *n* = 25	Pleomorphic Adenoma *n* = 25	*p*-Value (<0.05) *
Gender			0.8621
Female	9 F	16 F	
Male	16 M	9 M	
Age (years)	mean 67.7	mean 54.2	0.0032 *
BMI ^1^	27.5	25.3	0.4427
Normal weight	6	7	0.4832
Overweight	14	13	
Obesity	5	4	
Metabolic syndrome	0	1	
Smoking status “yes”	18/25	6/25	0.006 *
Duration smoking (years)	mean 17.2	mean 9	0.04131 *
Number of cigarettes per day	14.5	6.5	0.0064 *
Alcohol drinking			0.9655
<30 j/week	19/25	13/25	
>30 j/week	2/25	1/25	
Diabetes			0.7236
Diabetes type 1	1/25	0/25	
Diabetes type 2	1/25	1/25	
Hypercholesterolemia	5/25	4/25	0.0542
Hypertension artery	4/25	3/25	0.1823

^1^ Abbreviations: n—number; * *p*-values < 0.05 were statistically significant; BMI—body mass index.

**Table 2:** Characteristic features of the tumors.

Analyzed Trait	Warthin Tumor *n* = 25	Pleomorphic Adenoma *n* = 25	*p*-Value (<0.05) *
Tumor location:			N/A
Parotid	25/25	24/25	
Submandibular	0/25	1/25	
Sublingual	0/25	0/25	
Tumor size			0.7232
>1 cm	18/25	21/25	
<1 cm	7/25	4/25	
Type of surgery			N/A
Extracapsular dissection	0/25	0/25	
Partial parotidectomy	25/25	24/25	
Total parotidectomy	0/25	0/25	
Removal of submandibular gland	0/25	1/25	
The agreement between FNAB ^1^ results and postoperative histopathological diagnosis	81%	73%	N/A
Consistency of the tumor			0.4421
Soft	18/25	16/25	
Taut	3/25	7/25	
Not palpable	4/25	2/25	
Movable tumor in relation to the ground	21/25	23/25	N/A

^1^ Abbreviations: n—number; N/A—not applicable; * *p*-values < 0.05 were statistically significant; FNAB—fine-needle aspiration biopsy.

## Data Availability

The data that support the findings of this study are not openly available due to reasons of sensitivity and are available from the corresponding author upon reasonable request (mail: katarzyna.kolary@onet.pl).
